# Iatrogenic Cushing syndrome and adrenal insufficiency during concomitant therapy with ritonavir and fluticasone

**DOI:** 10.1186/s40064-015-1218-x

**Published:** 2015-08-27

**Authors:** Narendranath Epperla, Fergus McKiernan

**Affiliations:** Division of Hematology and Oncology, Medical College of Wisconsin, Milwaukee, WI USA; Center for Bone Disease, Marshfield Clinic, Marshfield, WI USA

**Keywords:** Ritonavir, Fluticasone, Cytochrome P450, Human immunodeficiency virus

## Abstract

Ritonavir is a potent inhibitor of the cytochrome P450 enzyme CYP3A4 and is subject to multiple drug–drug interactions. This becomes especially important when the patient is also taking medications metabolized through CYP3A pathway as increased and potentially toxic drug levels may ensue. Herein we present one such interaction wherein a 57 year old gentleman with human immunodeficiency virus (HIV) infection on highly active antiretroviral therapy that included ritonavir, had addition of fluticasone inhaler to his medication repertoire for treatment of chronic obstructive pulmonary disease. This resulted in severe osteoporosis, iatrogenic Cushing syndrome and adrenal insufficiency due to the potentiated systemic glucocorticoid effect of inhaled fluticasone by ritonavir. This case emphasizes the need for pharmacovigilance when managing patients on complex drug regimens for physicians treating HIV infected patients.

## Background

Ritonavir is a potent inhibitor of the cytochrome P450 enzyme CYP3A4 that can lead to multiple drug–drug interactions. Systemic complications resulting from inhaled corticosteroids like fluticasone are rare but when used concomitantly with ritonavir can lead to iatrogenic Cushing syndrome and adrenal suppression. It is important to be aware of this interaction to avoid serious and potentially fatal complications. We report a case of iatrogenic Cushing syndrome, adrenal insufficiency and severe osteoporosis due to the potentiated systemic glucocorticoid effect of inhaled fluticasone by ritonavir in a patient with human immunodeficiency virus (HIV).

## Case presentation

**Fig. 1:**
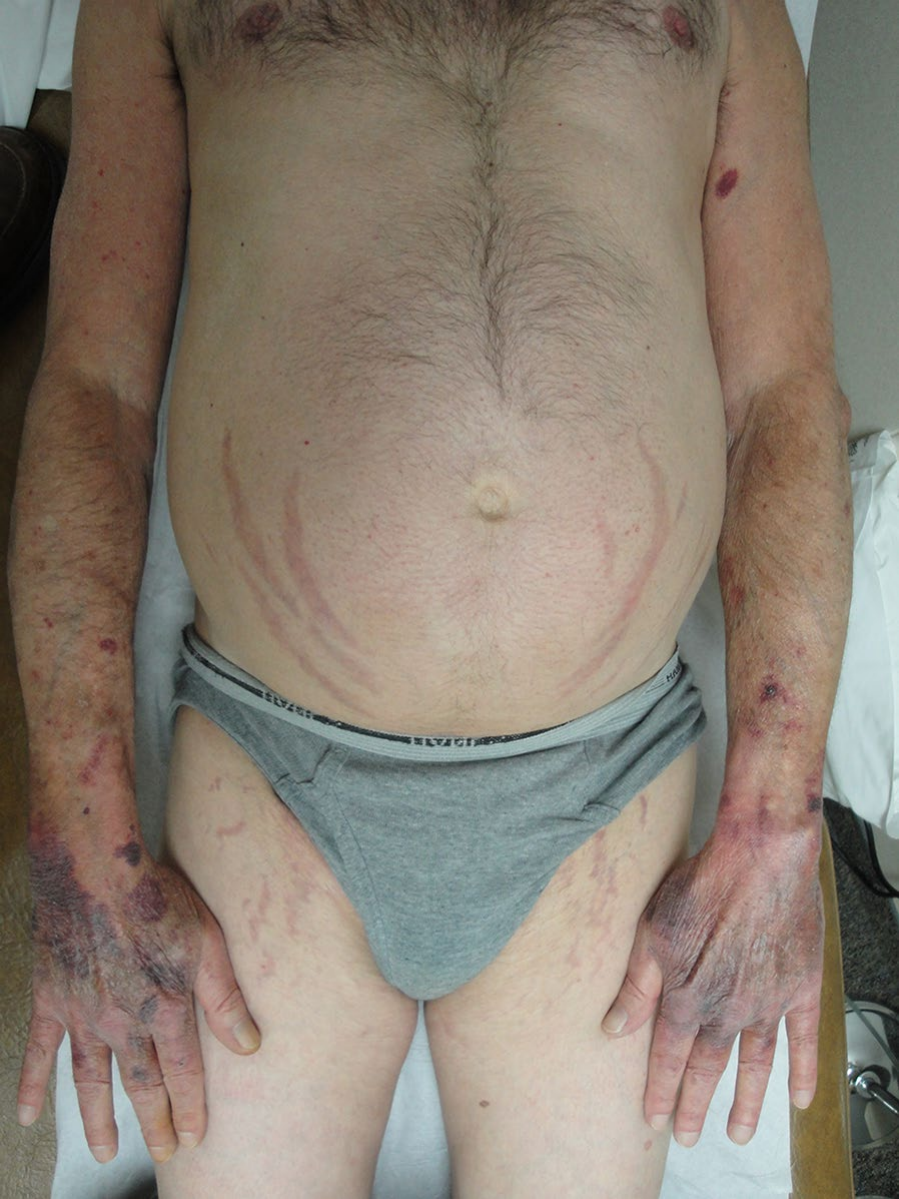
Centripetal adiposity, multiple ecchymoses and pronounced pink abdominal and inguinal striae

A 57 year old Caucasian male with HIV infection since 1986 was evaluated in 4/2010 for recurrent rib fractures following trivial stresses such as coughing. His highly active antiretroviral therapy (HAART) since 2006 consisted of lamivudine 150 mg twice daily, zidovudine 300 mg twice daily and lopinavir–ritonavir 400–100 mg twice daily. Fluticasone/salmeterol 250/50 mcg one puff twice daily was introduced in 9/2007 for severe COPD. Other medical conditions included coronary artery disease, dyslipidemia and GERD treated with aspirin, clopidogrel, omeprazole, pravastatin and niacin. Physical examination showed centripetal adiposity, multiple ecchymoses and pronounced pink abdominal and inguinal striae (Fig. 1). Weight had increased by 5 kg from baseline weight. There was palpable rib tenderness. The reminder of the physical examination was unremarkable. Of note his AIDS clinician mistook his phenotypic changes to be those of AIDS related lipodystrophy. These same changes eluded his PCP.

**Fig. 2:**
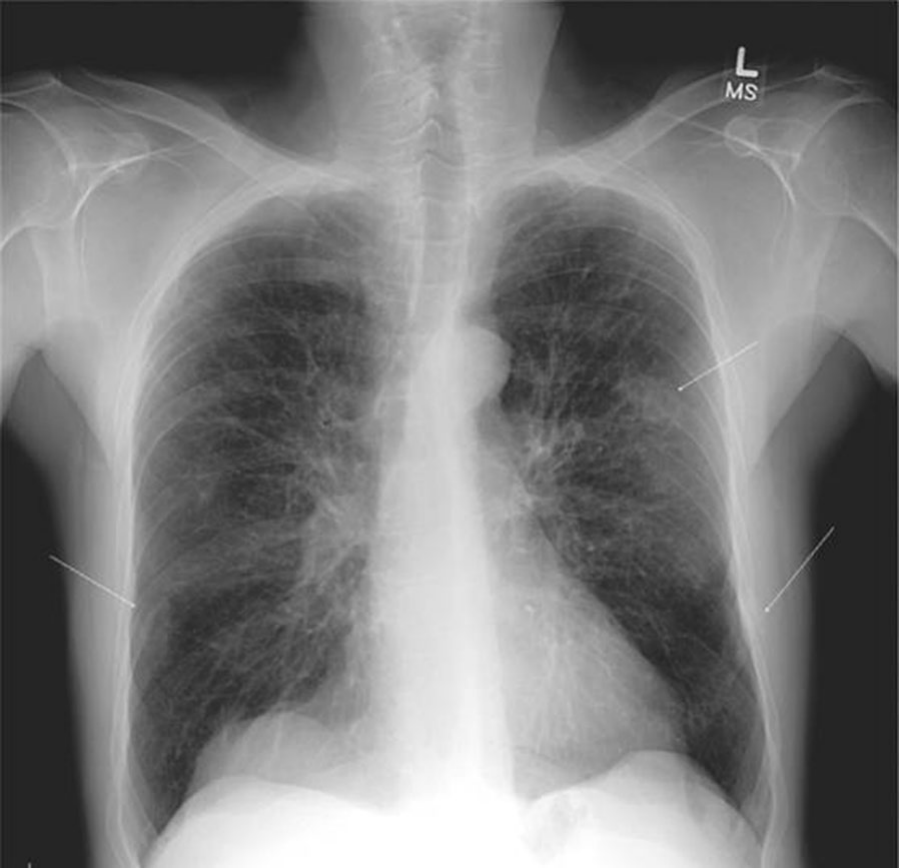
Chest X-ray showing numerous rib fractures

Chest radiographs confirmed numerous rib fractures (Fig. 2). Lumbar and proximal femur bone mineral density (BMD) T-scores were −5.2 and −3.4 respectively. Complete blood count, renal and hepatic function, electrolytes, calcium, phosphate, intact parathyroid hormone, 25-OH-VitD, prolactin, serum and urine protein electrophoresis and serum free light chains were normal. His random morning serum cortisol was 0.5 mcg/dl (normal 4–24 mcg/dL) and 1 h after 250 mcg intravenous cosyntropin stimulation was 7.1 mcg/dL (expected >20 mcg/dL) consistent with adrenal insufficiency. A 24 h urine free cortisol was <7.2 mcg, late night salivary cortisol was <10 ng/dL (<100 ng/dL) and dehydroepiandrosterone sulphate was <30 mcg/dL (40–310 mcg/dL). Serum ACTH was 32 pg/mL (normal 0–46 pg/mL). A 24 h urinary synthetic glucocorticoid screen was only positive for a fluticasone 17-β-carboxylic acid value of 243 pg/mL. Pituitary magnetic resonance imaging (MRI) was normal. Serum and 24-h urine N-telopeptides were 19.2 nmol BCE (5.4–24 nmol BCE) and 23 nmol/mmol BCE (21–66 nmol/mmol BCE) respectively. Total and bone specific alkaline phosphatase were 73 IU/L (20–71 IU/L) and 151 IU/L (40–125 IU/L) respectively.

The patient was diagnosed with iatrogenic Cushing syndrome and adrenal suppression secondary to the potentiated systemic glucocorticoid effect of inhaled fluticasone by ritonavir. Fluticasone was continued as the patient reported significant improvement in his COPD symptoms. Ritonavir boosted protease inhibitor therapy was discontinued and he was placed on Raltegravir which is not known to inhibit CYP3A4. His HIV viral load continues to be undetectable and 2 years after change in antiretroviral therapy his CD4 count is 624. He was placed on physiological doses of hydrocortisone at 20 mg per day and has gradually tapered to 5 mg/day of hydrocortisone 1 year from presentation. His severe osteoporosis was treated with subcutaneous teriparatide, appropriate calcium and vitamin D supplementation and a physical therapy.

**Fig. 3:**
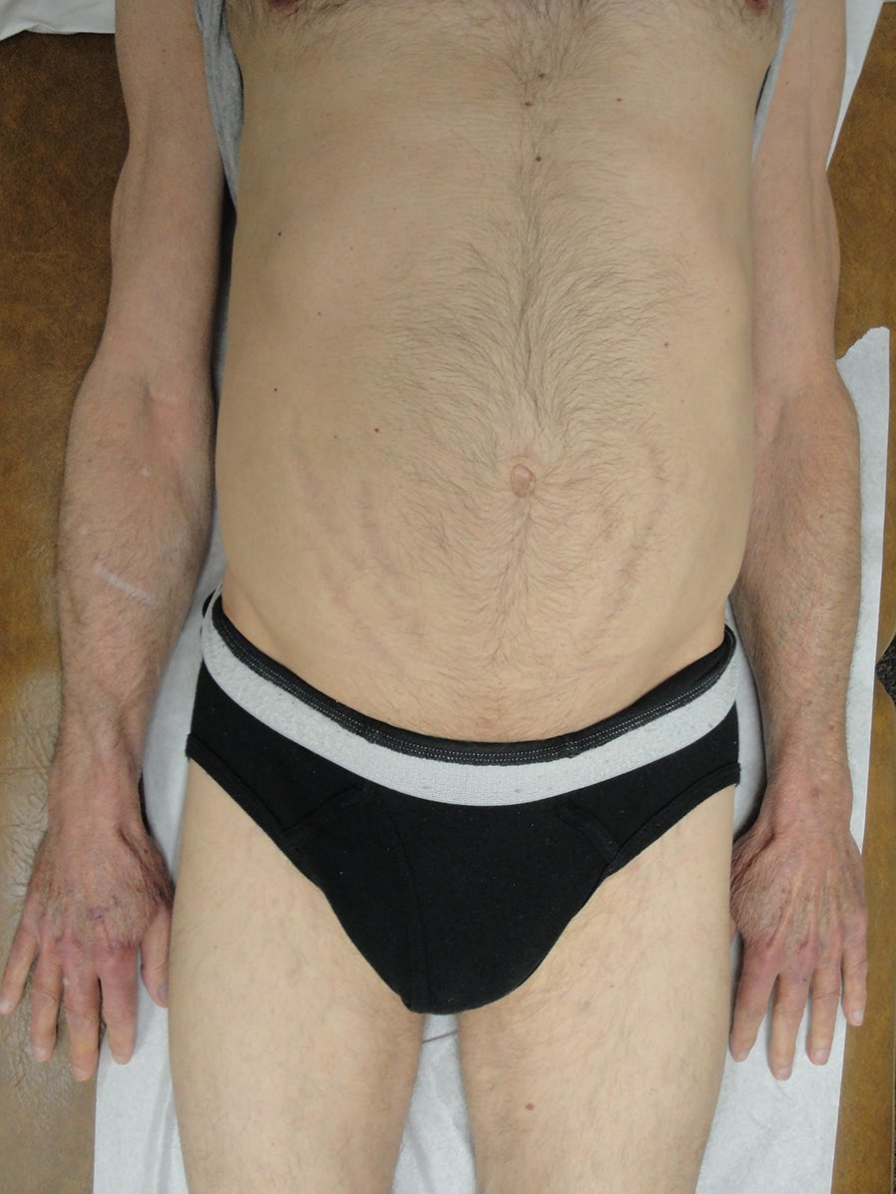
Markedly improved abdominal and inguinal striae as well as the ecchymotic lesions

**Fig. 4:**
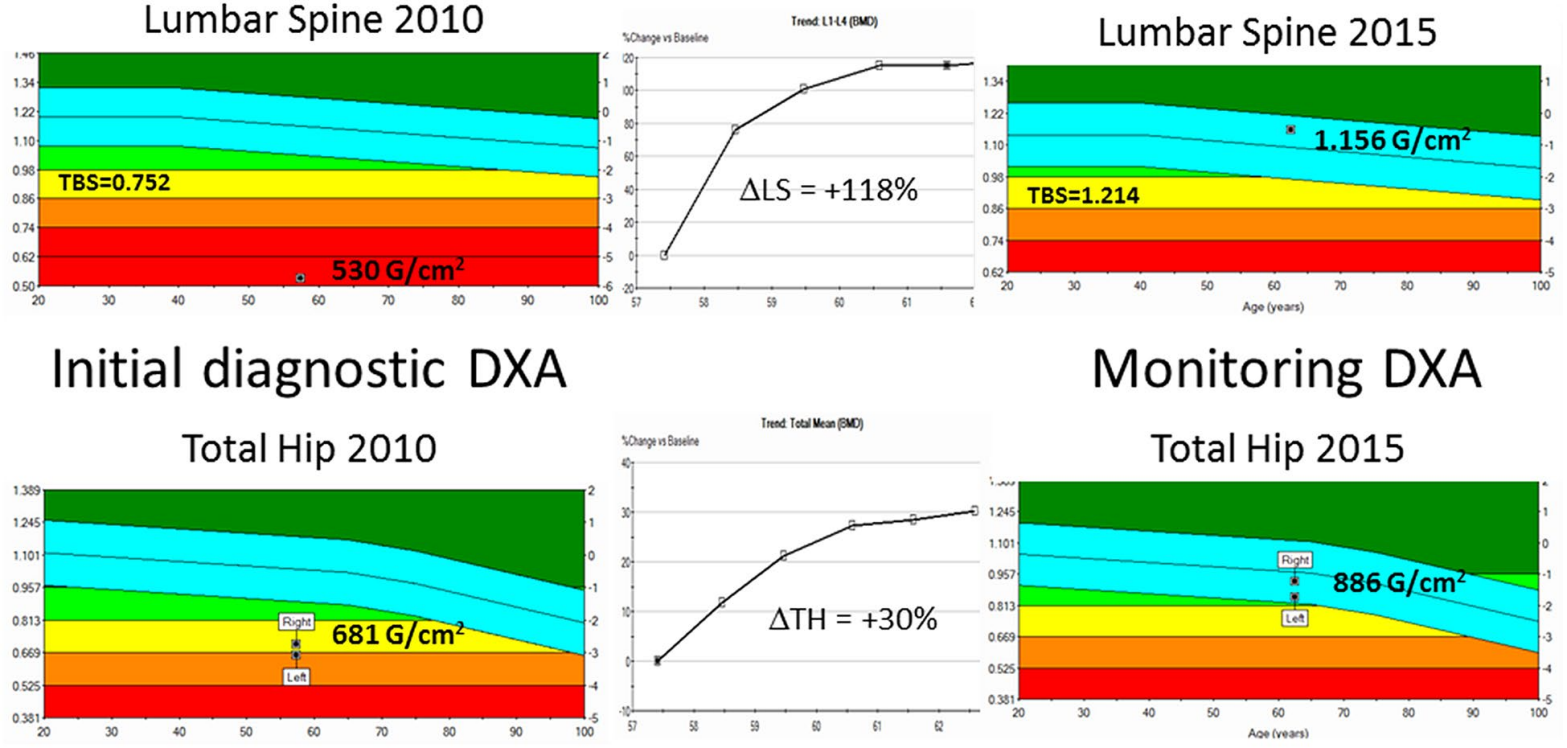
DXA trend after withdrawal of ritonavir and treatment with teriparatide along with depiction of improved trabecular bone scores

Serial basal and stimulated cortisol levels are reported in Table 1. His cushingoid features improved by 3 months (Fig. 3). One year after initiating osteoporosis treatment lumbar spine BMD improved from 0.530 to 0.932 gm/cm^2^ (+75.8 %) and total hip BMD from 0.681 to 0.761 gm/cm_2_ (+11.2 %). These BMD gains are significantly greater than those reported for lumbar spine (11 %) and total hip (5.2 %) in patients with glucocorticoid induced osteoporosis treated with teriparatide (Saag et al. 2009). Follow up BMD at the completion of teriparatide treatment is shown in Table 2 and Fig. 4. The patient has had no further fractures.

**Table 1:** Basal and stimulated cortisol levels

	Basal cortisol (mcg/dL)	1 h post ACTH cortisol level (mcg/dL)
At diagnosis	0.5	7.1
2 months	2.5	–
9 months	3.3	18
12 months	8.3	23.6
24 months	7	22.5

**Table 2:** BMD improvement after withdrawal of ritonavir and treatment with teriparatide

	BMD in gm/cm^2^ (T-score)		
	L-Spine	Total hip (mean)	Femoral neck (mean)
At diagnosis	0.530 (−5.7)	0.681 (−2.9)	0.589 (−3.7)
12 months	0.932 (−2.4)	0.761 (−2.4)	0.694 (−2.9)
24 months	1.065 (−1.3)	0.825 (−1.9)	0.734 (−2.6)
36 months	1.139 (−0.7)	0.867 (−1.6)	0.767 (−2.3)

## Discussion

A search of published English language literature using the keywords ritonavir, protease inhibitors, fluticasone, inhaled corticosteroids, Cushing’s syndrome and adrenal suppression revealed a total of 11 pediatric and 26 adult cases of iatrogenic Cushing’s syndrome and adrenal suppression from concomitant fluticasone and ritonavir therapy. 3 of 24 adult cases were secondary to intranasal fluticasone preparations and rest was from inhaled fluticasone. Fluticasone dose ranged from 500 to 2000 mcg/day in adult patients and 200–1000 mcg/day in pediatric patients. Ritonavir in both low doses “boosted” and in high doses was associated with significant interaction (Table 3).

**Table 3:** Review of literature and showing the cases of iatrogenic Cushing’s syndrome and adrenal suppression from concomitant fluticasone and ritonavir therapy

Authors	Age	RTV dose (mg/day)	ICS dose (mcg/day)	Duration^a^	Intervention
Mahlab-Guri and Asher (2011)					
Case 1	12 F	200	Fluticasone 200	6 months	Fluticasone replaced by montelukast
Case 2	55 F	200	Fluticasone 1000	3 weeks	Fluticasone replaced by budesonide
Case 3	65 F	200	Fluticasone 500	6 months	Fluticasone discontinued
Kaviani et al. (2011)					
Case 1	60 M	NA	Fluticasone 1200	2 years	Fluticasone discontinued
Kedem et al. (2010)					
Case 1	F	NA	NA	UNK	Fluticasone and budesonide discontinued
					Ritonavir dose decreased
Samaras et al. (2005)					
Case 1	43 M	200	Fluticasone 500	2 years	Fluticasone discontinued
Case 2	43 M	100	Fluticasone 1000	18 months	Fluticasone discontinued
Case 3	53 M	200	Fluticasone 1000	2 years	Fluticasone discontinued
Case 4	49 M	200	Fluticasone 1000	6 weeks	PI discontinued
Case 5	43 M	200	Fluticasone 500	4 months	Fluticasone discontinued
Case 6	50 M	100	Fluticasone 1000	2 months	Fluticasone discontinued
Nocent et al. (2004)					
Case 1	38 M	NA	Fluticasone 2000	1.5 months	Fluticasone replaced with beclomethasone
Leitman et al. (2009)					
Case 1	49 M	NA	Fluticasone 1000	UNK	Fluticasone dose reduced
Valin et al. (2009)					
Case 1	65 F	NA	Fluticasone 500	6 months	Fluticasone discontinued
Case 2	66 M	NA	Fluticasone 500	1 month	Fluticasone discontinued
Case 3	66 M	NA	Fluticasone 500	2 years	Fluticasone discontinued
Case 4	29 M	NA	Fluticasone 2000	1 month	Fluticasone discontinued PI replaced with nelfinavir
Jinno and Goshima (2008)					
Case 1	60 M	NA	Fluticasone NA	18 months	Fluticasone discontinued
Gillett et al. (2005)					
Case 1	27 F	200	Fluticasone 1000	10 weeks	Fluticasone discontinued
Soldatos et al. (2005)					
Case 1	66 M	200	Fluticasone 1000	4 months	Fluticasone dose decreased
Case 2	66 M	200	Fluticasone 1000	6 months	Fluticasone replaced with budesonide
Rouanet et al. (2003)					
Case 1	44 M	266.4	Fluticasone 2000	2 months	Fluticasone discontinued
Gupta and Dubé (2002)					
Case 1	45 M	800	Fluticasone 880	5 months	Fluticasone tapered
Clevenbergh et al. (2002)					
Case 1	33 M	200	Fluticasone 1000	5 months	Fluticasone discontinued
Chen et al. (1999)					
Case 1	32 M		Fluticasone 400	5 months	Fluticasone discontinued
Case 2	39 M		Fluticasone 800	18 months	Fluticasone discontinued
Hillebrand-Haverkort et al. (1999)					
Case 1	30 M	1200	Fluticasone 200	5 months	RTV replaced by NVP
Arrington-sanders et al. (2006)					
Case 1	11.4 F	133	Fluticasone 220		Fluticasone and PI discontinued
Case 2	20.9 M	100	Fluticasone 200		Fluticasone discontinued
Case 3	16.8 M	200	Fluticasone 250		Stopped all medications
Case 4	9.5 M	133	Fluticasone 220		Fluticasone discontinued
Case 5	1.8 F	60	Fluticasone 220		Fluticasone discontinued
Johnson et al. (2006)					
Case 1	12 F	134	Fluticasone 500	2 months	RTV/LPV D/C
Case 2	15 F	200	Fluticasone 1000	13 weeks	Fluticasone dose decreased
Pessanha et al. (2007)					
Case 1	16 F	NA	Fluticasone 500	3 months	RTV replaced by EFV
St Germain et al. (2007)					
Case 1	14 F	100	Fluticasone 500	2 weeks	Fluticasone discontinued
					RTV/ATV with held
Bhumbra et al. (2007)					
Case 1	9 M	108	Fluticasone 440	2 months	Fluticasone discontinued
			Mometasone 100	11 months	Mometasone discontinued
le Roux et al. (2001)					
Case 1	47 M		Budesonide 1600		Budesonide discontinued
Sagir et al. (2002)					
Case 1	48 M		Budesonide 9000	19 days	Budesonide discontinued

*RTV* ritonavir, *LPV* lopinavir, *ICS* inhaled corticosteroids, *EFV* efavirenz, *PI* protease inhibitors, *ATV* atazanavir, *NA* not available, *UNK* unknown, *NVP* nevirapine

^a^Duration until onset of first symptoms

Combination anti-retroviral therapy, particularly the introduction of protease inhibitors has revolutionized HIV therapy and changed once a fatal disease to a chronic condition (Palella et al. 1998). Ritonavir is a potent inhibitor of cytochrome P450 (CYP) 3A4 isozymes and significantly increases the concentration of drugs primarily eliminated by CYP3A metabolism such as macrolides, azoles, protease inhibitors and corticosteroids (Hsu et al. 1998; Von Moltke et al. 1998). This property of ritonavir is used to therapeutic advantage in ritonavir boosted protease inhibitor regimens and has decreased the pill burden and treatment failures and improved compliance with therapy (Thompson et al. 2010).

With 30 % estimated prevalence of bronchial hyperactivity (Poirier et al. 2001), HIV infected men who smoke are frequently exposed to inhaled corticosteroid therapy. To reduce airway inflammation treatment guidelines for asthma and chronic obstructive airway disease (COPD) recommend the routine use of inhaled corticosteroid alone or in combination with long acting bronchodilators (National Asthma Education and Prevention Program 2002; Vestbo et al. 2013). Fluticasone is a potent glucocorticoid commonly used in reactive airway disease. Compared with other available inhaled steroids, it has high glucocorticoid receptor binding affinity, is highly lipophilic, a large volume of distribution (318 L) (Wuerthwein et al. 1992; Mackie et al. 1996) and a longer elimination half-life (t_1/2_ 7–8 h). Less than 1 % of swallowed fluticasone is bioavailable due to its high first pass metabolism and rapid metabolism in liver by CYP3A4 enzyme system and conversion to inactive 17 B-carboxylic acid derivative (Harding 1990). Concomitant use of fluticasone with potent CYP3A4 inhibitors such as ritonavir can lead to systemic accumulation of fluticasone and suppression of hypothalamic pituitary adrenal (HPA) axis. Ritonavir increased the area under concentration–time curve (AUC) of serum fluticasone by 350-fold in healthy volunteers (Laboratories 2006). For this reason manufactures and the FDA recommend against routine use of combination of ritonavir and fluticasone unless benefits outweigh risks.

Fluticasone has been reported to cause greater dose related adrenal suppression when compared with budesonide, triamcinolone acetonide or beclomethasone dipropionate (Lipworth 1999) even in the absence of CYP3A4 inhibitors like ritonavir. In a recent meta-analysis of 732 subjects with asthma, fluticasone in small to medium doses (50–500 mcg/day) alone showed minimal effect on adrenal function (Masoli et al. 2006). In another prospective, non-randomized, open-label, cross sectional study, investigators found that patients taking high doses of fluticasone (>880 mcg per day) for longer duration had abnormal adrenal function (White et al. 2006).

Iatrogenic Cushing syndrome results from prolonged exposure to high doses of glucocorticoids. The vast majority of these cases result from administration of oral or parenteral glucocorticoids (Newell-Price et al. 2006). Typical features include weight gain, central obesity, dorsocervical hump, moon face, facial plethora, thin skin, easy bruising, abdominal striae, hirsutism, proximal myopathy, osteopenia, glucose intolerance, hypertension, nephrolithiasis and psychiatric manifestations such as depression and psychosis (Newell-Price et al. 2006). Osteoporosis is common and tends to involve trabecular bone resulting in an increased risk of fracture within 3 months of daily exposure (van Staa et al. 2000, 2002, 2005).

Pituitary production of corticotropin (ACTH) will be suppressed by exogenous steroids, which leads to atrophy of the adrenal cortex and adrenal insufficiency. Diagnosis is confirmed by low early morning serum cortisol levels and subnormal response to standard ACTH stimulation test. An early morning serum cortisol levels <3 mcg/dL (80 nmol/L) strongly suggests adrenal insufficiency, whereas levels >15 mcg/dL (415 nmol/L) predicts a normal response of serum cortisol to ACTH stimulation test (Hagg et al. 1987; Le Roux et al. 2002). Patients with low or equivocal serum cortisol levels in whom adrenal insufficiency is suspected should undergo standard or low dose synthetic ACTH (Cosyntropin) stimulation test. Suboptimal response to Cosyntropin stimulation test is diagnostic of adrenal insufficiency. Simultaneous measurement of plasma ACTH helps in differentiation of primary from secondary or tertiary adrenal insufficiency. ACTH levels tend to be higher than normal in primary adrenal insufficiency whereas low or low normal in patients with secondary and tertiary adrenal insufficiency. Corticotropin-releasing hormone (CRH) test further differentiate secondary from tertiary adrenal insufficiency (Schulte et al. 1984).

Human immunodeficiency virus associated lipodystrophy shares several morphological features and should be differentiated from Cushing Syndrome. Weight gain, central adiposity, dorsocervical hump, insulin resistance, osteopenia, dyslipidemia are common and are associated with ART therapy (Lichtenstein 2005; Dube et al. 2007; Carr and Cooper 1998). Presence of facial plethora, cutaneous striae, and proximal myopathy differentiates it from Cushing syndrome and should prompt physicians to evaluate for HPA axis suppression. Sudden withdrawal of from steroid therapy has a potential to develop catastrophic adrenal crisis. Replacement with physiological doses of prednisone 5–7.5 mg a day, hydrocortisone 15–20 mg a day or equivalent should be initiated. Measurement of morning cortisol levels every 4–6 weeks serves as screening test for recovery of adrenal function. Morning serum cortisol level less than 3 mcg/dL indicates the need for continued replacement therapy, whereas a value greater than 20 mcg/dL indicates recovered HPA axis. Patients with morning cortisol levels between 3 and 20 mcg/dL will need further studies like Cosyntropin stimulation test or overnight metyrapone test. It may take 9–12 months for the recovery of adrenal function (Hopkins and Leinung 2005).

In patients with iatrogenic Cushing syndrome and adrenal insufficiency secondary to the interaction between ritonavir and fluticasone available options include, replacing ritonavir with another antiretroviral agent, replacing fluticasone with another less potent steroid or leukotriene antagonists or long acting anticholinergic agent such as tiotropium. In the SPIRAL study, Raltegravir demonstrated the non-inferior efficacy and improved lipid profile when ritonavir boosted protease inhibitor therapy was replaced by Raltegravir (Martinez et al. 2010). The latter approach was taken in our patient with favourable outcome.

## Conclusions

Despite the recommendations against concomitant use of fluticasone and ritonavir several reports of Cushing syndrome and adrenal suppression are being reported. We believe physicians treating HIV infected patients must be aware of potential interaction of antiretroviral therapy and drugs used to treat associated co-morbidities. When iatrogenic Cushing syndrome is suspected, prompt evaluation and discontinuation of offending medication will prevent potentially fatal complications.

## Consent

Written informed consent was obtained from the patient for publication of this case report and any accompanying images. A copy of the written consent is available for review by the Editor-in-Chief of this journal.

## Abbreviations

HIV: human immunodeficiency virus
CYP: cytochrome P450
BMD: bone mineral density
HAART: highly active antiretroviral therapy
